# Indents

**Published:** 1917-02

**Authors:** 


### INDENTS

☞ **Brief Articles of Technical or Scientific Value will receive notice and comment. Contributions invited.**

*Focal Infections.* For many years it was thought that we were well acquainted with bacteria and their vagaries, but recently we have discovered that there remains much to be learned. Of the various activities of bacteria the most interesting at present is that of the so-called focal infection. By this is meant a small, more or less quiescent point of disease which, although it causes no local disturbances, gives rise to symptoms elsewhere.

Probably the most important of these manifestations is the involvement of the various joints of the body.

At present the capable physician is no longer content to give anti-pyretics, in expectation of a miraculous cure. If a patient now gives a history of chronic and painful joints, the first thought of his physician should be focal infection. To determine the presence or absence of such a condition is not always an easy task, and outside aid, particularly the Röntgen ray, may have to be called upon. There may be a chronic gonorrhea, the tonsils may be diseased or, what is very common, there may be infection at the roots of the teeth. This last is a frequent condition and may be present without local indications. It is also interesting to note that many inflammatory lesions of the eye are directly referable to dental infections. This has long been recognized by the laity, but the idea was considered to be mere superstition by the medical high priests.

After removal of the focal infection the patient's rapid recovery, in many instances, is little short of marvelous. In most cases no further treatment seems to be necessary, the joints cease to be painful, and the convalescent goes on his way rejoicing.—*New York Medical Journal.*

*The Search for the Ideal Antiseptic.* The ideal antiseptic procedure obviously would be one which would destroy the infecting micro-organisms but leave undamaged the body cells with which it might come into contact. The quest for such a substance has continued with enthusiasm almost since the time of Koch's fundamental researches on chemical disinfectants published thirty-five years ago. The substances proposed during this period have been legion. They represent the most diverse types of chemical compounds, acting by a variety of reactions, but all resulting in a damage to the bacterial body, whether it be by rapid oxidation, by coagulation of the protoplasm, by dehydration, by chemical combination or by some more subtle process. The living body is a structure of living cells comparable in many respects with the inimical micro-organisms. The tissue cells, therefore, are likely to experience as much damage from efficient antiseptics as do the invading bacteria. Experience teaches what considerations of comparative biology lead one to expect, that in infected wounds, for example, the antiseptic must often be applied in such strength that body cells as well as bacteria are injured or destroyed. Nevertheless, the search for the ideal has continued in the hope, often seemingly useless and unjustified, of ultimately finding some discriminating agent suitable for bacterial destruction. After all, the tissue cells are not identical with the lowest forms of microbiotic structure, or entirely analogous to them. One technical difficulty in the planning of investigations has been the lack of suitable isolated body cells which could be observed with respect to the possible effects of antiseptics. This has been overcome, however, by the use of so-called tissue cultures in which the growth of isolated connective tissue cells can now easily be watched in the laboratory. In this way R. A. Lambert has followed the effect of the same chemical agents on bacteria and tissue cells growing together in vitro. Some estimate of the comparative resistance of body tissues to various antiseptic chemicals should thus be gained. It is apparent from his study that in the case of the chemical substances tested, such as potassium cyanid, phenol (carbolic acid), tricresol, hydrogen dioxid and alcohol, tissue cells were definitely more easily killed than were bacteria. With certain other disinfectants the difference was not so striking. Iodin stands out as the one chemical tested to which cells were found more resistant than were staphylococci. A good growth of cells was seen after exposure to a 1:2,000 solution of iodin for an hour, a strength sufficient to sterilize the tissue completely in most instances. Lambert points to his experiments as further evidence of the widely accepted value of iodin as an antiseptic. They indicate that, at least in weak aqueous solution, it should not, as is often stated, injure or irritate the tissues. Lambert is careful to point out, however, that iodin has the power of rapidly dissolving fibrin. Ordinarily the union of wound surfaces by the fibrin facilitates healing. The use of fibrin-dissolving, but tissue-conserving solutions raises a debatable theoretical question which application in practice can perhaps best decide.—*Journal A. M. A.*, January 6, 1917.

*Some Conclusions as to Root Treatments.* If you are really desirous of perfecting your work in this branch of dentistry, select a lot of extracted teeth or roots; open them, clean out the canals, working until you have passed through the apical foramen, and then fill them. If you are unable to do this satisfactorily with teeth out of the mouth, what percentage of success will you have with teeth in the mouth? Join a study club, get together and not only watch the other fellow, but get busy yourself.

I feel that after working on teeth out of the mouth, noting the large percentage of abnormal roots of which the difficulty of cleansing and filling is only appreciated when attempted, and from the discoveries of research workers and radiologists that is absolutely essential that this work be properly performed, we have been brought to a keen realization of our inefficiency in dealing with the problem. That, although we can not coincide with all their views, when men have practiced a definite, systematic technique in any branch of dentistry for twenty-five years with a large percentage of successes, we are greatly indebted to them, and their methods are entitled to our most thoughtful consideration.

That the pulp of a tooth is never to be devitalized unless absolutely necessary.

That we shoud realize that the opening, cleansing and filling of root canals in a scientific manner is comparable to the most delicate operations in surgery and must be carried on in a surgical manner. That if such experts as Rhein, Grieves, Ottolengui, Best, and others can successfully fill ninety per cent. of teeth, the average practitioner will do well with sixty-five or seventy-five per cent., but as Ottolengui says, the more canals men *try* to fill, the more canals *will* be filled.

That in sodium-potassium and sulfuric acid we have valuable but dangerous agents, requiring great care in their use lest we bring about a condition equally as bad as that which we are endeavoring to eradicate.

That while radiography is of incalculable value, it is not an infallible guide and the making and interpretation of radiographs is a study in itself.

That only through accurate data compiled from records kept for a series of years can we determine the most satisfactory technique.

That immunity and vital resistance play a larger part in the final outcome of our work than we now realize.

That taking pictures at stated intervals of devitalized teeth to determine the condition of the investing tissue is a wise procedure.

That for the physical welfare of our patients, it were far better to extract many of the abscessed teeth or those with unfillable roots instead of trying to save them. And above all—

That we require more than anything else an awakening of conscience that will not let us rest content with our work until it has been greatly improved.

That the dentist of the future must, in addition to his increased mechanical skill, be fortified with a profound knowledge of anatomy, histology, bacteriology and pathology, and be able to apply that knowledge, and finally—

That if we are to realize that degree of tooth conservation so much desired, both he of the present and he of the future must bend every effort in assisting in the great wave of education regarding oral prophylaxis going on throughout the world. Thus may we contribute our share toward the betterment of humanity and help in the solution of the root-canal problem.—Dr. B. Boyd, in *Pacific Gazette.*

*Investing and Casting Gold Inlays.* There are many good investment materials on the market, and my only reason for giving the formula of that which I use is the fact that it can be made a good deal more cheaply than bought, and to most of us that is an object. We take one part of plaster of Paris, three parts of finely pulverized silex and one-half part of Dixon's flake graphite. This is thoroughly mixed together by sifting it through an ordinary flour sifter three or four times. I always aim to use the same quantities of the investment material and water, and in order to do so, always measure them. If we are to have definite results we must have definite methods. We are aiming to keep all of the processes at room temperature, so we use water that is about one hundred to one hundred and twenty degrees in temperature, according to the temperature of the room, for if we use water that is just the right temperature we will find that the cold bowl, cold plaster, etc., will make the investment too cold, while the water being a little warmer than necessary will obtain a mixture that is just about right without the bother of complex paraphernalia to obtain the same result.

Before the model is coated with the investing material, I wash it off with a little soap on a camel hair brush to remove any oily or muciferous materials that would prevent the perfect adaptation of the plaster to the wax. The model is now coated with the soft investment material, carefully excluding any bubbles and the investment completed in the flask. As soon as the investment is hard I begin to burn out, and am very careful that this is not overdone. More inlays are spoiled by reason of the overheating of the investment than any other way, for the plaster is disintegrated and made weak by too much heat and under the pressure of casting, gives ground to the force of the pressure and a distorted misfitting inlay is the result. I have recently installed a one heat electric heater that is giving the greatest satisfaction. I leave the moist flask—that is, before the moisture has been driven off—on the heater that has had the current on for about a minute and then turned off, for five minutes, when I know that the investment is dry and is ready for the heat. We then turn on the current and leave it on for ten minutes, after which the electricity is turned off and the flask set aside to cool. Every operator will have to determine just the right time by experimenting with his heater, for they will not all be the same and the current may differ in different places. The investment will appear dark, for the wax is not entirely evaporated from it, but it makes no difference, as the casting made will be better for not heating the flask too much. I cast with the flask cold. In doing so I believe that I obtain better results. In fact, we can heat the flask to such a degree that the inlay will be so expanded that it will not go into the cavity; and by chilling the wax and heating the flask we can obtain a fairly good compensation in the expansion of the gold for the shrinkage of the wax. That method, however, is uncertain and I believe unscientific, therefore I attempt to follow all operations at the same temperature and not try to make up at one end for the fault of the other. I use the Taggart machine in casting and believe that it is the best in every respect, though good results may be obtained with other machines. The gold should be melted with the oxy-hydrogen blow-pipe because of the quickness of the effect produced. If the ordinary blow-pipe is used the entire flask will be heated up again and it will be impossible to cast cold.

The gold should be brought to a white heat, but should not boil and if the boiling point is reached, it is well to hesitate a moment before applying the pressure or there will be a liability of failure in the casting. A force of five pounds is sufficient to cast and a much greater pressure is contraindicated, for the reason that it will be liable to break or distort the investment. The pressure should be maintained for at least thirty seconds in order to force all of the gold in the mold that is possible, for Dr. Price's experiments demonstrate that up to a certain point in the freezing, that more gold can be forced in the mold to take the place of the shrinkage. I use 23-K. gold in all cases where the inlay is subject to masticatory stress; and in proximal cavities in incisors and cavities in all of the gingival surfaces, I use pure gold, for here there will be no stress and the pure gold will answer the purpose; but wherever there is any degree of strain, pure gold is contraindicated, for it is too soft and will flow under the stress of mastication. When the inlay has cooled we remove from the flask, wash and pickle in hydrofluoric acid for a few moments to remove all traces of calcarious matter.—Dr. Conzett, in *Journal of Allied Societies.*

*The Ameba Buccalis in Pyorrhea Alveolaris.* Dr. Joseph Mendel, of Paris, has recently completed in the laboratory of Dr. Saliembeni, in the Pasteur Institute, a series of investigations on the rôle of the ameba buccalis which we report editorially because of the interest awakened in this country by the investigations of Smith and Barrett and Bass and Johns. He says in the *Annali di Odontologia* that he has not restricted his studies to the rôle of the ameba in pyorrhea alveolaris. He has studied the morphology of the organism and the significance of its presence or absence in pyorrhea alveolaris; in mouths free from pyorrhea alveolaris; in acute and chronic alveolar abscesses; in acute gingivo-stomatitis; in abscesses caused by the eruption of the third molar and in dental caries. He also reports on the success of the treatment of pyorrhea alveolaris with emetine hydrochlorid. In the course of the work forty pyorrhea cases were examined in individuals ranging from nineteen to sixty-five years of age, the majority of whom were sufferers from gastro-intestinal disturbances, namely, dyspepsia, constipation, enteritis, entero-colitis, etc.

Clinically the cases varied from a slight lesion in one or two teeth accompanied by a very moderate discharge to a profound anatomo-pathologic lesion covering a relatively large number of teeth and causing an abundance of purulent discharge capable of producing grave disturbances in the patient's general health. All the suppurations were of gingival origin. The ameba was present in thirty-eight out of the forty cases and in each instance in sufficient numbers to make the examination an easy task. Every drop of pus secured did not contain ameba, but whenever obtained from the deepest portion of the pocket the ameba was present. In a series of forty-two adults and thirty-six children absolutely free from pyorrhea alveolaris, ranging in age from twenty to forty years and from eight months to fourteen years the ameba was present in fifty-seven per cent. of the adults and in twenty-two per cent of the children. It is to be noted that in those cases free from pyorrhea alveolaris, reported as positive, the ameba was present in sufficient numbers to wipe out the element of doubt, from the standpoint of any possible degree of pathogenic action. Had the author considered it advisable to report every case in which the ameba was present even in very small numbers the percentages would be markedly increased. In twelve cases of acute alveolar abscess, the ameba was absent; in twelve cases of chronic alveolar abscess the ameba was present. In other words, the ameba was absent in acute infections and was present in fairly large numbers in chronic infections. Concerning treatment with emetine hydrochloride by instillations into pyorrhea pockets or by intra-gingival injections the author's results were negative. Emetine in his opinion is not a cure for pyorrhea alveolaris. The conclusions reached by the author are as follows:

1. The ameba buccalis is present in practically every case of pyorrhea alveolaris.

2. The ameba is always present in the slimy material which coats the teeth.

3. The presence of the ameba in the mouth is not *pathognomonic* of pyorrhea alveolaris. It is not present exclusively in connection with that disease.

4. The ameba buccalis is present in at least fifty per cent. of clean mouths. Its presence coincides with a more or less marked decrease in vital resistance and with a hyper-leucocytosis in the gingival exudate; it could be considered as a symptom of a predisposition to pyorrhea alveolaris.

5. The ameba was absent in the acute infections of the mouth; on the contrary its presence was frequently observed in the chronic infections. The ameba plays no rôle whatsoever in the process of dental caries.

6. The treatment of pyorrhea alveolaris by instillations or injections of emetine hydrochloride proved to be a failure in every case tried by the author.

7. Systematic oral prophylaxis is the surest means of entirely eliminating the ameba or at least of greatly decreasing its number.—Editorial in January *Pacific Gazette.*

*Examples of a Dietary that Will Not Produce Caries, and One that Will.*

"We may illustrate the two different types of diet which are often more or less consistently followed, by the following dietaries:

"First, we shall refer to the kind of meal which does not produce caries:

"*Breakfast*—Fish, bacon, toast and butter, fruit, coffee, and tea. (For children:—milk, water.)

"*Luncheon*—Meat or poultry, potatoes, salad, baked bread, pudding, fresh fruit, water.

"*Supper*—Rusks, toast, or bread rolls and butter, chicken or fish, an apple, tea or coffee. (For children:—water, milk.)

"Secondly, we may outline the kind of meal which induces dental caries:

"*Breakfast*—Porridge and milk, bread and marmalade. Then perhaps a supplementary breakfast a few hours after, of a glass of milk and a sweet biscuit.

"*Luncheon*—Mashed potatoes, and gravy, or minced meat, milk and pudding.

"*Supper*—Bread soaked in milk, or bread and jam, cocoa and cake, and a supplementary supper on going to bed, of a glass of milk and a biscuit, or just 'a tiny piece of chocolate.'

"On comparing these two different types of diet, we observe that one is of a kind which stimulates mastication and the last thing taken leaves the mouth clean, or at least free from carbohydrates, so that even when soft food is part of the meal the mouth will be physiologically clean at the end of the meal. The other type is intended to represent the kind of meal which is calculated to lodge about the teeth, and to ruin them within a few years by making efficient mastication and the self-cleansing of the mouth practically impossible, and by leaving the mouth sticky with fermentable carbohydrates and a virulent crop of acid-forming micro-organisms which have had their development encouraged by the previous meal."—Dr. A. W. Crosby, in *Cosmos*.

*Important Information Regarding the Dental Protective Association of the United States.*

The annual meeting of the Dental Protective Association of the United States, was held at the Palmer House, Chicago, Illinois, on December 18, 1916, at four p. m. A brief synopsis of the meeting is here given for the benefit of the members who were unable to attend in person.

The treasurer, Dr. D. M. Gallie, reported that the total assets of the Association were $35,617.04. Of this amount, $26,000 is in approved municipal bonds, $4,000 in Harbough notes, secured by collateral notes, as reported at our last annual meeting, and the balance in cash in a savings and checking account.

It was reported last year that Dr. A. F. James, of Chicago, a member of the Association in good standing, had been sued by Cassius M. Carr for an alleged infringement of a patent. Since this time Dr. Thomas B. Hartzell, of Minneapolis, also a member of the Association in good standing, has been sued by the same party, and on practically the same grounds. We quote from the report of the attorney for the Association, Mr. P. B. Eckhart, of the law firm of West and Eckhart, regarding these suits, both of which are being defended by the Association: "During the past year your attorney's activities for the Association have been for the most part, directed toward the preparation for trial of the two patent suits brought against members of the Association. These cases are now ready for trial. One of them will likely be disposed of without trial so far as the Association's interest in it is concerned. On Tuesday, December 12th, the United States court sustained our motion to dismiss the bill for lack of jurisdiction. Upon the merits of the case we expect to be able to defeat the claim of the patentee. It is interesting to note that a decree by default against an eastern dentist, who had neglected to procure an attorney or pay any attention to the suit, was entered in a patent suit brought in Chicago by the same claimant. This decree can not be used as a precedent against us, however, and it is doubtful whether it has any validity by reason of certain defects in procedure."

A matter of importance to the members is an intended change to be made in the by-laws of the Association by the Board of Directors. According to the by-laws it has been necessary to mail a notice of the annual meeting to each individual member. Inasmuch as there are no annual dues to be collected, and inasmuch, also, as only a few members attend the annual meeting in person, it seems to be a needless expense and entails unnecessary labor on the part of the officers, who are not compensated for their services, to send this individual notice. This matter was discussed and it was agreed by all present that a notice in the Journal of the National Dental Association, and all other important journals, should be sufficient; that it was generally understood that the annual meeting of the Association is always held on the third Monday in December, at four p. m., in Chicago. The Board of Directors accordingly will make this change in the by-laws, and all members should take notice and govern themselves accordingly.

The work of the Board of Directors for the past year was ratified by the Association by a unanimous vote; and the present Board was re-elected for the ensuing year.

By order of the Board of Directors: J. G. Reid, President; J. P. Buckley, Vice-President and Secretary; D. M. Gallie, Treasurer.

*Ethyl Chlorid Spray for Pain.* For pain when the tissues have not been lacerated spray the part affected until the frost begins to form, and repeat several times if necessary. If there is a wound, spray around and as near the laceration as practicable without spraying the injured part, as it would be painful to get the drug on the abraded tissue. If there are sensitive teeth involved they can be protected by wax or modling compound. When the effect is produced it remains for considerable time, as it is not transient like local anesthesia.—Dr. F. W. Stephan, in *Dental Review.*

